# Improving time to TAVI with a local TAVI clinic: A single-centre quality improvement project

**DOI:** 10.1016/j.fhj.2026.100537

**Published:** 2026-04-29

**Authors:** Ravi Chotalia, Mohammed El-tayeb, Jan Kovac, Elved Roberts, Kazi Adnan, Juan Fernandez, Vinod Venugopal

**Affiliations:** a Lincolnshire Heart Centre, Lincoln County Hospital, United Lincolnshire Hospitals NHS Trust, Lincoln, UK; b Ross Lucas Medical Sciences Building, College of Health and Science, University of Lincoln, Lincoln, UK; c Cardiology Department, Glenfield Hospital, Groby Road, Leicester, UK; d Department of Cardiovascular Sciences, University of Leicester, Leicester, UK

**Keywords:** Aortic stenosis, TAVI, Time interval, Delivery of healthcare, Rural patients, TAVI pathway, Hospitalisations

## Abstract

**Introduction:**

Delays to transcatheter aortic valve implantation (TAVI) carry significant morbidity and mortality, with patients referred from non-tertiary centres experiencing significantly longer waiting times.

**Methods:**

A local TAVI clinic was established in January 2024. Data were collected from 2021 to 2024. The primary outcome was time from referral to TAVI. Comparisons were made between our centre, the tertiary centre, another referring centre with a dedicated TAVI pathway, and centres without dedicated clinics. Secondary outcomes included aortic stenosis-related hospitalisation.

**Results:**

Median time to TAVI significantly decreased following clinic establishment: 218 days pre-clinic vs 77 days post-clinic, p < 0.0001. Time to TAVI was comparable to tertiary and other dedicated TAVI-pathway centres and shorter than referral centres without dedicated clinics. Aortic tenosis-related hospitalisations were significantly lower: nine (12.5%) pre-clinic vs five (4.0%) post-clinic, p = 0.025.

**Conclusion:**

The introduction of a local TAVI clinic in our centre was associated with reduced time to TAVI and fewer aortic stenosis-related hospitalisations.

## Highlights

• Delay to TAVI carries significant morbidity and mortality.
• Patients from non-tertiary centres experience significantly greater delay to TAVI compared to patients from tertiary centres.
• Establishment of a local TAVI clinic in non-TAVI centre significantly reduced the median time to TAVI from 218 days to 77 days.
• Time to TAVI was comparable to tertiary and other dedicated centres and shorter than centres without dedicated clinics.
• A local TAVI clinic was associated with fewer patients experiencing an aortic stenosis-related hospitalisation.

## Introduction

Aortic stenosis (AS) is the most common valvular heart disease, and as the population continues to age, the prevalence of severe AS is expected to double by 2050.^1^ Severe, symptomatic AS has a poor prognosis without treatment, with a 50% mortality at 2 years.^2^

Transcatheter aortic valve implantation (TAVI) has emerged as an effective and safe treatment option for severe AS, improving mortality, hospitalisations and quality of life.^2^ European Society of Cardiology guidelines now recommend TAVI over surgical aortic valve replacement in patients above 70 years of age if their anatomy is suitable.^3^

In the UK, there is a demand for TAVI that outstrips its supply, with only 50% of TAVI centres consistently able to meet the British Cardiovascular Intervention Society (BCIS) treatment target of 18 weeks (<126 days).^4^, ^5^ There is also significant geographical inequality in TAVI and SAVR (surgical aortic valve replacement) provision, with patients in rural areas experiencing greater delay and reduced access both in the UK and in the USA.^6^, ^7^ Moreover, patients who are referred from a non-TAVI centre face significantly longer delays to TAVI than those managed at an on-site TAVI centre, which are typically located in urban areas.^8^ Delay in treatment of severe AS is costly, resulting in increased mortality, increased frailty, and increased peri-procedural complications.^7^ Wijeysundera *et al* found that patients who waited greater than 180 days had a wait-time mortality of 28.9%, vs a 1.9% wait-time mortality if TAVI was performed within 10 days.^9^ Moreover, Roule *et al* have found that each week's delay from referral to TAVI increases 1-year mortality by 2%.^10^ Studies have also found that increasing waiting times are associated with higher rates of heart failure hospitalisations.^11^

In recent years, novel hub-and-spoke model TAVI clinics hosted at non-TAVI centres prior to referral have been shown to reduce wait times for patients undergoing TAVI^12^ and have been associated with fewer reported adverse events in abstract-level analyses,^13^ compared to previous referral pathways. These novel pathways have also been shown to improve overall pathway efficiency compared with neighbouring non-TAVI centres over the same time period.

The primary aim of this quality improvement project was to assess the impact of a similar local TAVI clinic focusing on comprehensive local work-up including clinical assessment, counselling and cardiac computed tomography (CCTA) on time to TAVI. We also aimed to compare this time interval with that experienced by patients at the tertiary centre, a comparison not reported previously,^12^ as well as the corresponding time intervals at neighbouring referral centres. A secondary aim was to assess whether implementation of the pathway was associated with a reduction in proportion of patients experiencing aortic stenosis-related hospitalisations in the pre-procedural period.

## Methods

### Details of the local TAVI clinic

**Fig. 1:**
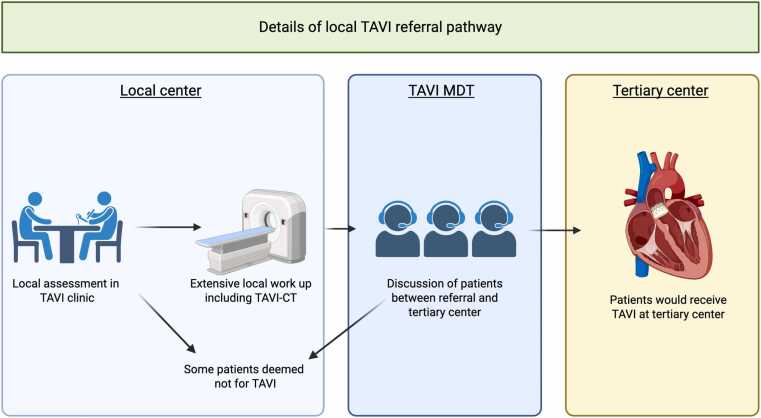
A figure representing details of our local TAVI referral pathway. Created with Biorender.com and have approval to use. Abbreviations: MDT, Multidisiciplinary team meeting.

**Fig. 2:**
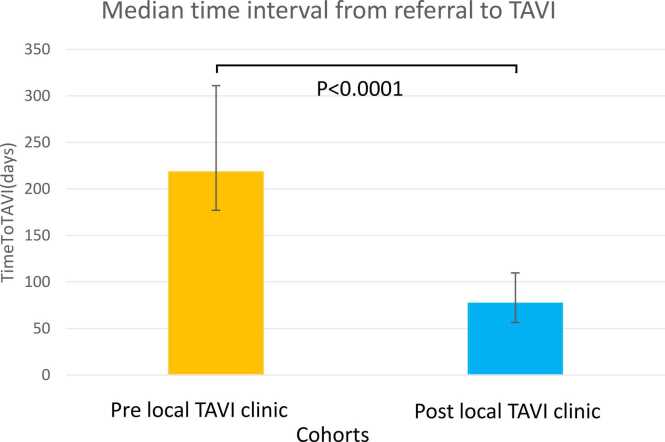
Median time to TAVI before and after establishment of local TAVI clinic. Error bars represent the interquartile range. The line above the bars is a significance bar to represent the significance difference between values.

Commenced in January 2024, the TAVI clinic was set up as a dedicated structural clinic in our non-tertiary centre (United Lincolnshire Teaching Hospitals). The clinic was staffed by local cardiologists with prior structural training alongside a dedicated TAVI specialist nurse, providing comprehensive clinical assessment for patients referred for TAVI. Assessment included evaluation of symptoms, comorbidities, frailty, quality of life and patient expectations to determine suitability for intervention. If suitable, patients were counselled for the procedure and given information leaflets. They were then listed for a gated CCTA performed within 2 weeks, paying particular attention to pre-hydration and stopping of nephrotoxic medications due to the risk of contrast-induced nephropathy in this cohort (see Fig. 1, Fig. 2). Invasive coronary angiography was not performed as standard. In certain cases, confirmatory tests such dobutamine stress echocardiography or aortic valve calcium scoring were required. If deemed suitable, a referral was made to the tertiary centre with the date of the planned CCTA.

The CCTA was reported by both local cardiologists and tertiary centre radiologists and subsequently reviewed at the TAVI MDT (multidisiciplinary team) meeting. MDT discussions were attended by the local cardiologists and TAVI nurses within the TAVI clinic, alongside operators from the tertiary centre, during which cases were reviewed in detail. Patients deemed unsuitable or unfit for TAVI at MDT review were not listed for the procedure and continued under conservative management by the local TAVI team. Where transfemoral suitability was confirmed, patients were listed directly for the procedure. Cases with alternative access requirements, or where suitability remained uncertain following MDT review, required a dedicated face-to-face review at the TAVI centre by the surgical or high-risk anaesthetic teams prior to final procedural decision.

In the pre-TAVI clinic era, patients were assessed through standard referral pathways via general cardiology outpatient clinics, with evaluation performed by consultant cardiologists without prior structural training. Imaging and onward referral for TAVI were arranged on an ad hoc basis, with all patients needing face-to-face review at the TAVI centre.

### Data collection

Data were collected for all patients who underwent a transfemoral TAVI procedure under local anaesthetic via an outpatient pathway. Patients undergoing TAVI via alternative access routes (eg subclavian or transaortic), those requiring general anaesthesia, and those who received a TAVI as an inpatient were excluded.

Data were prospectively recorded from initiation of the TAVI clinic in January 2024 until November 2025. From October 2021 to January 2024, the regional TAVI centre provided retrospective data on patients from our centre who received a TAVI.

All data were obtained from electronic medical records. Baseline demographic data, comorbidities and echo parameters were recorded, along with the date of referral to either the TAVI centre or local TAVI clinic, the date of MDT and the date of the TAVI procedure.

The regional TAVI centre also provided data on referral dates, MDT dates and TAVI procedure dates for patients from that centre and from neighbouring local hospitals in the post-TAVI clinic period from January 2024 until August 2025. At the time of initiation of our local TAVI clinic, a neighbouring hospital, referral centre 1, had also set up a dedicated TAVI pathway.

The study was reported according to the SQUIRE 2.0 guidelines.^14^ The study was approved by the institutional audit department of United Lincolnshire Teaching Hospitals NHS Trust.

### Outcomes

The primary outcome of this study was the median time to TAVI, defined as the number of days from referral to the date the patient underwent a TAVI procedure. This was compared between the pre-TAVI clinic and post-TAVI clinic periods at our centre. Time to TAVI in the post-TAVI clinic period (January 2024–November 2025) was also compared with that of the regional TAVI centre, referral centre 1, and other neighbouring local hospitals without local TAVI clinics over the same period.

We additionally assessed the proportion of patients receiving a TAVI within 18 weeks (<126 days) and after 180 days and calculated the associated odds ratios. These proportions were compared between the pre- and post-TAVI clinic periods at our centre.

### Aortic stenosis-related hospitalisations

In addition, data were collected on the proportion of patients with at least one aortic stenosis-related hospitalisation during the pre-procedural waiting period, defined as a hospital admission directly attributable to complications or progression of aortic stenosis. Qualifying events included decompensated heart failure, pre-syncope, syncope or chest pain considered secondary to aortic stenosis. Hospitalisations deemed unrelated to aortic stenosis were not included within this definition. This included episodes of chest pain treated as acute coronary syndrome secondary to acute plaque rupture, major bleeding events and all other non-aortic stenosis-related hospital admissions. Mortality could not be compared as, in the pre-TAVI clinic era, data were only available for patients who received a TAVI.

### Referral pathway outcomes

In our centre, we also recorded data on the proportion of patients referred to the TAVI clinic who were subsequently referred for a TAVI, as well as those who were not referred and the reasons for this in the post-TAVI clinic era. As referral pathway data from the pre-TAVI clinic period were not prospectively collected and the regional TAVI centre only provided data on the patients who ultimately received a TAVI, we were unable to compare the referral pathway outcomes from the pre- and post-TAVI clinic periods.

### Statistical analysis

Results are presented as medians (interquartile range (IQR)). Data were assessed for normality using the Shapiro–Wilk test. Chi-squared test was used to compare proportions between groups and, where small cell counts (<5) existed, Fisher’s exact test was used. Non-parametric continuous variables were compared with the Mann–Whitney *U* test or the Kruskal–Wallis test, with post-hoc Dunn testing and Bonferroni correction where appropriate. Aortic stenosis-related hospitalisations were a post-hoc analysis and reported as raw numbers and proportions and compared between groups using chi-squared test and Fisher’s exact test. To account for differences in follow-up time between the two groups, total event rates were also calculated as event rates per patient-week of follow-up and compared between groups using Poisson regression analysis. Significance was defined as p < 0.05. All statistical analysis was performed using Stata (v18.0, StataCorp, College Station, TX).

## Results

### Demographics

Between October 2021 and December 2023, prior to the local TAVI clinic, 72 patients underwent TAVI after referral. From January 2024 to November 2025, after the TAVI clinic was set up, 125 patients underwent TAVI after referral. The median age of patients in the overall cohort was 83 years (IQR 79.3–86.8) and 35% of patients were female. There was no significant difference in patient demographics pre- and post-TAVI clinic set up (Table 1).

**Table 1:** Baseline characteristics of patients in the pre-TAVI clinic and post-TAVI clinic eras. Continuous variables are reported as median (interquartile range) unless otherwise stated.

Characteristic	Pre-TAVI clinic cohort (n = 72)	Post-TAVI clinic cohort (n = 125)	p-value^1,2,3^
Age at time of TAVI	81 (78–85)	84 (81–87)	0.45
Female sex, n (%)	24 (33.3%)	45 (36.0%)	0.70
**Comorbidities**			
COPD, n (%)	15 (20.8%)	25 (20.0%)	0.32
Diabetes, n (%)	32 (44.4%)	49 (39.2%)	0.81
Previous myocardial infarction, n (%)	15 (20.8%)	28 (22.4%)	0.8
Hypertension, n (%)	56 (77.8%)	100 (80%)	0.71
Chronic kidney disease^a^, n (%)	38 (52.8%)	63 (50.4%)	0.75
eGFR mL/min/1.73 m^2^	60 (55–72)	58 (48–70)	0.22
Prior cardiac surgery, n (%)	12 (16.7%)	25 (20.0%)	0.21
Anaemia±, n (%)	27 (37.2%)	50 (40.0%)	0.40
Atrial fibrillation, n (%)	20 (27.8%)	28 (22.4%)	0.40
History of cancer n (%)	23 (31.9%)	32 (25.6%)	0.34
**Left ventricular (LV) ejection fraction, n (%)**			
Preserved LV function: ≥50%	48 (66.7%)	88 (70.4%)	0.42
Mildly impaired LV function: 40–49%	9 (12.5%)	13 (10.4%)	
Moderately impaired LV function: 35–39%	8 (11.1%)	12 (9.6%)	
Severely impaired LV function: <35%	7 (9.7%)	12 (9.6%)	
**Aortic valve echo parameters**			
Aortic valve area (cm^2^)	0.81 (0.72–0.92)	0.75 (0.65–0.9)	0.27
Peak aortic valve gradient (mmHg)	72 (58.7–78.4)	75 (62.5–84.1)	0.58
Mean aortic valve gradient (mmHg)	42 (32.1–52.1)	44 (34.9–53.0)	0.70
Dimensionless velocity index	0.20 (0.17–0.23)	0.22 (0.19–0.24)	0.79

Abbreviations: COPD, chronic obstructive pulmonary disease; eGFR, estimated glomerular filtration rate.

1,2,3 Analysed using chi-squared test, Mann–Whitney *U* test or Kruskal–Wallis test.

a Defined as an eGFR <60 mL/min/1.73 m^2^ ±defined as a haemoglobin <10 g/L.

### Time to TAVI

The median time from clinician referral to TAVI pre-TAVI clinic was 218 days (IQR 171–311.5), vs 77 days (IQR 115.5–168.75) post-TAVI clinic, p < 0.0001 (Fig. 1). This was driven by a decrease in median time from referral to MDT (153.5 days (82.5–225) vs 40.5 days (24–70) p < 0.0001) as well as time from MDT to TAVI (68 days (43.7–126) vs 32 days (22–43) p < 0.0001), suggesting improvements in both the efficiency in local TAVI assessment and improvements in tertiary centre capacity (Table 2).

**Table 2:** Time to TAVI outcomes in patients referred for TAVI from our centre, pre- and post-TAVI clinic establishment.

	Pre-TAVI clinic (n = 72)	Post-TAVI clinic (n = 125)	p-value^1,2,3^
Median time to TAVI, days	218 (171–311.5)	77 (58–119)	<0.0001
*Median time from referral to MDT, days*	153.5 (82.5–224)	40.5 (24–70)	<0.0001
*Median time from MDT to TAVI, days*	68 (43.7–126)	32 (22–43)	<0.0001
Time to TAVI <126 days, n (%)	10 (13.9%)	95 (76%)	<0.0001
Time to TAVI >180 days, n (%)	51 (71%)	11 (8.8%)	<0.0001

Abbreviations: MDT, Multidisciplinary team.

1,2,3 Analysed using chi-squared test, Mann–Whitney *U* test or Kruskal–Wallis test.

The number of patients waiting more than 180 days from referral to TAVI was 51 (71%) pre-TAVI clinic vs 11 (8.8%) post-TAVI clinic (odds ratio (OR) 0.04 (95% confidence intervals 0.02–0.09), p < 0.0001), while the number of patients who received a TAVI within 126 days (the BCIS recommendation) was 10 (13.9%) vs 95 (76%) p < 0.0001 (OR 19.6 (95% CI 9.0–43.0), p < 0.0001).

### Aortic stenosis-related hospitalisations

The establishment of our local TAVI clinic was associated with a reduction in the proportion of patients experiencing aortic stenosis-related hospitalisation (nine patients (12.5%) pre-TAVI clinic to five patients (4.0%) post-TAVI clinic, p = 0.025) (Table 3). There was no significant difference in the event rate per patient week compared between the groups: 0.0035 events per patient week pre-TAVI clinic vs 0.0029 events per patient week post-TAVI clinic (incidence rate ratio 0.81 (0.27–2.42), p = 0.7).

**Table 3:** Number of patients experiencing an aortic-stenosis-related hospitalisation, and causes of hospitalisation, pre- and post-TAVI clinic establishment.

	Pre-TAVI clinic (n = 72)	Post-TAVI clinic (n = 125)	p-value^1,2,3^
≥1 aortic stenosis-related hospitalisation, n (%)	9 (12.5%)	5 (4.0%)	0.025
Causes of hospitalisation			
Decompensated heart failure, n (%)	2 (2.8%)	1 (0.8%)	0.55
Chest pain attributable to aortic stenosis, n (%)	3 (4.2%)	2 (1.6%)	0.36
Presyncope/syncope, n (%)	4 (5.6%)	2 (1.6%)	0.19
Event rate (total events per patient-week)	0.0035	0.0029	0.7

Abbreviations: MDT, Multidisciplinary team.

1,2,3 Analysed using chi-squared test, Fisher's exact test or Poisson regression analysis.

### Referral pathway outcomes in local TAVI clinic

Between January 2024 and November 2025, a total of 240 patients were referral to the local TAVI clinic. A total of 150 (62.5%) were listed for TAVI after discussion with the TAVI centre, of whom 124 had received their TAVI at the time of analysis. 20 (8.3%) patients were deemed unfit for TAVI due to frailty or comorbidities conferring prohibitive procedural risk or limited expected benefit, while five (2.1%) patients were deemed unsuitable due to anatomical or technical factors precluding safe TAVI implantation. Both groups were recommended palliative/medical treatment. 16 (6.7%) patients became acutely unwell and were transferred as an inpatient; six (2.5%) patients were referred for SAVR; 15 (6.2%) patients were asymptomatic and were recommended a watch and wait strategy; eight (3.3%) patients died awaiting intervention; one (0.4%) patient was deemed not to have severe AS after further investigation and eight (3.3%) patients were awaiting further investigations to either confirm severe AS or investigate comorbidities which could also have been contributing to symptoms. After further discussion at clinic, 10 patients (4.1%) declined the procedure. In the pre-TAVI clinic era, there were only data for patients who received a TAVI.

### Time to TAVI vs. other centres

**Fig. 3:**
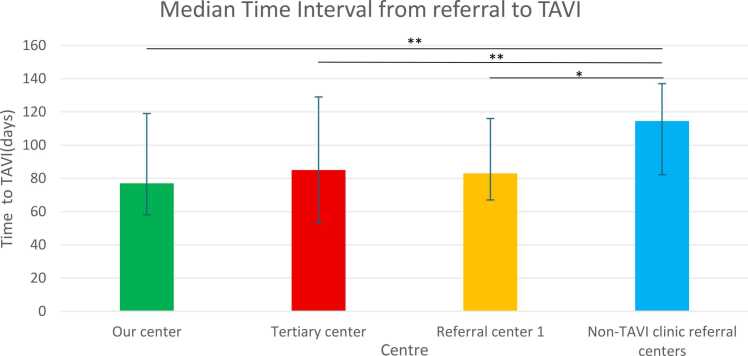
A bar chart demonstrating median time to TAVI in our centre compared to other centres. Error bars represent the interquartile rate across the median value. The lines above the bars are significance bars to represent significance differences between values. *= p < 0.05 ** p < 0.01.

There was no significant difference in the median time to TAVI in the post-TAVI clinic era between our centre (median time 77 days (IQR 58–119), the tertiary centre (median time 82 days (IQR 53–129) p = 0.99 and referral centre 1 (which also has a local TAVI clinic) (median time 83 days (IQR 67–116) p = 1.00. However, there were significant differences between all three centres and the median of other regional hospitals where there was no local TAVI clinic set up (median time 115 days (IQR 81–139) p < 0.05) (Fig. 3).

## Discussion

In this single-centre quality improvement project, we demonstrated that implementation of a local TAVI clinic in a non-surgical rural centre was able to significantly reduce the time from referral to TAVI. Median referral-to-TAVI time decreased from 218 days pre-clinic to 77 days post-clinic (p < 0.0001). Moreover, the proportion of patients waiting more than 180 days from referral to TAVI fell substantially, while the proportion meeting the BCIS wait-time target of <126 days increased. Importantly, post-implementation waiting times were comparable to those seen at the tertiary centre and another referring centre with a dedicated local TAVI clinic, and significantly lower than the time experienced by patients at neighbouring centres without a dedicated local TAVI clinic.

These findings are clinically important, as studies have identified that delays to TAVI increase wait-time mortality and risk of hospitalisation,^11^ reduce symptom improvement post-TAVI and increase peri-procedural complications.^7^, ^9^, ^10^

Consistent with this, although event numbers were small, establishment of a dedicated TAVI clinic was also associated with a significant reduction in the proportion of patients experiencing aortic stenosis-related hospitalisation in the pre-procedural period: from nine patients (12.5%) to five (4.0%) in the post-implementation era (p = 0.025). This is likely driven by reduced waiting times, as there was no significant difference in event rates when adjusted for patient follow-up time. However, mortality could not be compared in our study due to limitations in our dataset in the pre-TAVI clinic era, as discussed previously. Future studies, ideally with larger sample sizes, are required to more robustly evaluate the impact of dedicated TAVI clinic implementation on hospitalisation and mortality outcomes.

An additional benefit of the pathway was improved patient selection. Studies have identified that those with significant frailty do not have frailty reversed by TAVI and do not receive the same mortality or quality-of-life benefits as non-frail patients^15^, ^16^ and the benefit of TAVI in asymptomatic patients is an area of ongoing research and has not been conclusively established.^17^ 90 of the 240 patients referred to the local TAVI clinic were not ultimately referred/listed for a TAVI due to being unfit, having unsuitable anatomy or being asymptomatic. This approach conserves tertiary care resources and reduces unnecessary lengthy travel for frail patients.

Factors which enabled us to establish a local TAVI clinic include the presence of consultants with prior structural training and interest, the support of a dedicated TAVI specialist nurse who coordinated the pathway, as well as a dedicated and well-established cardiac CT service. A highly accurate TAVI-CT is a prerequisite for TAVI: it is essential for assessing suitability of vascular access, predicting potential complications and sizing of TAVI valves.^18^ However, access to cardiac CT remains variable across the UK; lack of CT availability is recognised as a substantial issue by 45% of TAVI centres in the UK^19^ and there is significant geographic variation in its availability.^20^ Absence of this may limit other centres from adopting a similar pathway, and therefore investment into TAVI-CT availability is a key area where improvements should be sought to be implemented by non-tertiary centres.

These findings are consistent with those reported by Hewitson *et al*,^12^ who also evaluated a novel TAVI pathway with emphasis on extensive local work-up. They found that patients from their centre had significantly reduced wait to TAVI (32.4 vs 126 days) and a higher proportion of patients proceeding to TAVI (97.8% vs 49.5%), compared with centres without a dedicated local TAVI clinic. Building on this, we have also demonstrated that such a model can achieve wait times comparable to that of a tertiary centre. This finding is important, as previous studies^8^ have demonstrated that patients who are referred from a non-TAVI centre face longer delays to TAVI than those managed at a tertiary TAVI centre. Our results suggest that establishment of a dedicated TAVI clinic in a non-TAVI centre can reduce this recognised inequality in TAVI access.

## Limitations

Our data have several limitations. First, in the pre-TAVI clinic period, we only received data for patients who ultimately underwent a TAVI procedure. This limited our ability to compare the proportion of referred patients who ultimately were accepted for a TAVI, or mortality between the pre- and post-TAVI clinic periods. Second, all the improvements in median time to TAVI may not have solely been due to improvements in our pathway, as during the same period, there were also significant improvements in the procedural capacity of the tertiary centre. This is evidenced by a significant decrease in median time from MDT discussion to TAVI between the pre- and post-TAVI clinic periods. However, the reduction in median time from referral to MDT (113 days) was greater than the decrease in time from MDT to TAVI (36 days), suggesting much of the improvement in overall time to TAVI is likely due to improvements in our pathway. Finally, the findings from a regional TAVI referral network may not be directly generalisable to other healthcare systems.

## Conclusions

In conclusion, the establishment of a local TAVI clinic in our centre was associated with a reduction in time to TAVI and a lower proportion of patients experiencing aortic stenosis-related hospitalisation. Implementation of similar pathways in other centres may help to improve access to TAVI, where delay has been shown to carry significant morbidity and mortality.

## CRediT authorship contribution statement

**Ravi Chotalia:** Writing – review & editing, Writing – original draft, Methodology, Formal analysis, Data curation. **Juan Fernandez:** Writing – review & editing, Conceptualization. **Kazi Adnan:** Writing – review & editing, Methodology, Conceptualization. **Mohamed El-tayeb:** Writing – review & editing, Data curation. **Elved Roberts:** Writing – review & editing, Supervision, Methodology. **Jan Kovac:** Writing – review & editing, Supervision. **Vinod Venugopal:** Writing – review & editing, Writing – original draft, Supervision, Project administration, Methodology, Conceptualization.

## Ethics approval and consent to participate

The study was approved by the Institutional Audit Department of United Lincolnshire Teaching Hospitals NHS trust as part of quality improvement of the TAVI management pathway. Due to the nature of the study, participant consent was not required.

## Funding

This research did not receive any specific grant from funding agencies in the public, commercial, or not-for-profit sectors.

## Declaration of competing interest

The authors declare that they have no known competing financial interests or personal relationships that could have appeared to influence the work reported in this paper.

## Data Availability

The full data set (anonymised) is available upon reasonable request by contacting the corresponding author.
